# Comparative genomic analysis of *Campylobacter hepaticus* genomes associated with spotty liver disease, Georgia, United States

**DOI:** 10.3389/fmicb.2023.1215769

**Published:** 2023-06-29

**Authors:** Julia Ienes-Lima, Roel Becerra, Catherine M. Logue

**Affiliations:** Department of Population Health, College of Veterinary Medicine, University of Georgia, Athens, GA, United States

**Keywords:** *Campylobacter hepaticus*, poultry, genome, comparative analysis, *Campylobacter* spp.

## Abstract

*Campylobacter hepaticus* has re-emerged as an important cause of disease in egg laying birds worldwide, resulting in morbidity, mortality, and significant losses in eggs for the breeding and table egg laying industries. Although birds may appear asymptomatic, the disease is characterized by spots on the liver of birds and histopathological analysis reveals multifocal fibrogranulocytic necrotizing hepatitis microscopically. The re-emergence of *C. hepaticus* may be linked with housing practices as the disease appears more prevalent in pasture raised birds with outside exposure. Here we describe, the whole genome sequences and comparative analysis of four *C. hepaticus* genomes associated with an outbreak on pasture raised breeders from a farm in Georgia, United States. All four genomes were relatively similar in size and virulence genes harbored. Using these genomes, comparison with current *C. hepaticus* genomes available in NCBI and other databases and other members of the *Campylobacter* species was carried out. Using current tools available, virulence gene factor content was compared, and it was found that different tools lead to different numbers of factors identified. The four genomes from this study were relatively similar to *C. hepaticus* HV10 the type strain from Australia but differed from the other sequenced US strains from Iowa and Florida. *C. hepaticus* was found to have an overall lower gene content for genes associated with virulence and iron acquisition compared to other *Campylobacter* genomes and appears to cluster differently than UK genomes on phylogenetic analysis, suggesting the emergence of two lineages of *C. hepaticus*. This analysis provides valuable insight into the emerging pathogen *C. hepaticus*, its virulence factors and traits associated with disease in poultry production in the US, potentially providing insight into targets for its control and treatment for laying birds. Our analysis also confirms genes associated with iron acquisition are limited and the presence of the multidrug efflux pump CmeABC in *C. hepaticus* which may promote survival and persistence in the host niche – the chicken liver/bile. One unique aspect of this study was the finding of a close genetic relationship between *C. hepaticus* and *Campylobacter fetus* species and evidence of genome reduction in relation to host niche specificity.

## 1. Introduction

*Campylobacter hepaticus* has re-emerged as a pathogen of interest and a causative agent of spotty liver disease (SLD) in egg producing poultry worldwide. First recognized in the 1950s the disease was characterized as a “vibrio-like” organism with first reports in the United States and subsequent cases emerged in other regions of the world including the United Kingdom, Canada, Australia, New Zealand, and Europe (Crawshaw et al., 2015; Van et al., 2016; Van T. T. H. et al., 2017; Courtice et al., 2018; Crawshaw, 2019; Crawshaw et al., 2021). The disease disappeared for a number of decades and was likely linked to the cage systems used in modern poultry operations (Courtice et al., 2018; Gregory et al., 2018). With the re-emergence of pasture raised birds for the organic market and animal wellness issues, SLD appears to have once again emerged. Recent reports from Australia noted that the disease was common but seems to have increased in prevalence and reemergence (Phung et al., 2019; Van et al., 2019). Reports from the United States have also indicated the presence of the disease in laying birds in Iowa (Gregory et al., 2018; Wu et al., 2021), Florida (Arukha et al., 2021) and, most recently in Georgia (Becerra et al., 2023b). Anecdotal reports from the US seem to suggest there is a seasonal association between outbreaks of the disease and the warmer months.

In infected birds, *C. hepaticus* causes multiple white/gray spots on the livers as well as reductions in egg production and mortality (Crawshaw and Young, 2003; Grimes and Reece, 2011; Phung et al., 2019), in some cases, mortality as high as 10% has been reported with egg reductions of 25% (Crawshaw and Young, 2003; Grimes and Reece, 2011; Phung et al., 2019; Van et al., 2022). In addition, pre-disposition of a flock increases the likelihood of clinical disease usually in the form of a stressor (Van et al., 2022). Evidence suggests SLD is most common in free range layers, especially those free ranging outdoors or on deep litter (Crawshaw and Young, 2003) as opposed to caged layers where the disease does not appear to be as common; however, cases of SLD have also been reported in caged layers (Grimes and Reece, 2011; Gregory et al., 2018; Arukha et al., 2021). Outbreaks of SLD appear to be common in the summer months and may correlate with other previously named diseases including avian vibrionic hepatitis, avian infectious hepatitis, summer hepatitis and military hepatitis (Crawshaw and Young, 2003; Grimes and Reece, 2011; Crawshaw et al., 2015; Petrovska et al., 2017a,b; Courtice et al., 2018). Recent works have confirmed that *C. hepaticus* is the causative agent of SLD by experimentally replicating the disease in challenged birds and confirming Kochs postulates (Crawshaw et al., 2015; Van T. T. et al., 2017). In addition, the emergence of a second *Campylobacter* species also linked with SLD has recently been confirmed using Kochs postulates, and this organism has been identified as *Campylobacter bilis* (Van et al., 2022). The relationship between *C. bilis* and *C. hepaticus* as causative agents of SLD is the subject of ongoing research.

Histological analysis of the livers from SLD affected birds show random multifocal necrotic hepatitis sometimes with heterophil and macrophage infiltration (Crawshaw and Young, 2003; Becerra et al., 2023b). Additional analysis of livers from the Georgia case (from which the genomes in this study originated) reported mild to moderate subacute multifocal cholangiohepatitis with biliary hyperplasia and rupture as well as multifocal necrotizing and fibrinolymphocytic or heterohistiocytic hepatitis consistent with an ascending or hematogenous infection likely of bacterial origin (Becerra et al., 2023b).

To date, the genome sequences of *C. hepaticus* that are available for analysis and study are limited, which presents a challenge for identification of virulence traits and potential targets for control and vaccine development. Here, we analyzed the genomes of four *C. hepaticus* isolated from our study (Becerra et al., 2023a) and those available in NCBI and other genome databases, using the *C. hepaticus* type strain HV10 (Van et al., 2016) as reference genome. We also included an additional series of *Campylobacter* genomes from the genus that are closely related to compare our sequenced genomes with those of other *Campylobacter* species. All genomes were subjected to analysis for virulence traits, whole genome comparison and phylogenetic analysis using SNP analysis. This study provides new information on *C. hepaticus* genomes and shows the relationship between *C. hepaticus* and other members of the *Campylobacter* genus when compared at the whole genome level.

## 2. Materials and methods

### 2.1. Bacterial isolates

Four *C. hepaticus* strains were isolated from organic pasture raised laying hens in Georgia, United States, in 2021 (Becerra et al., 2023b). Bile samples were collected immediately post-mortem from different birds associated with the outbreak farm and showed the presence of spotty livers on necropsy. Volumes of the bile (30 μL) were plated directly on blood agar plates (TSA + 5% Sheep blood, Remel) and cultured under microaerophilic conditions (Mitsubishi, MicroAero Gas generator, Remel) in an anaerobic jar at 37°C for 7 days. Suspect colonies were picked and suspended in sterile water to make DNA using the boil prep method (10 min at 100°C, followed by cooling and centrifugation to extract the DNA). Confirmation was carried out by PCR targeting the glycerol kinase gene using primers and conditions previously described by Van and collaborators (Van T. T. H. et al., 2017).

### 2.2. Genome sequencing and analysis

Genomic DNA was extracted from each strain using the MagAttract HMW DNA Kit (Qiagen, Hilden, Germany). The DNA sample was quantified using Qubit^®^ dsDNA HS Assay Kit (Life Technologies, Carlsbad, CA), and the quality was evaluated using a NANODROP 2000 (ThermoFisher Scientific, Waltham, MA), followed by analysis using E-Gel SizeSelect 2% Agarose Gel (Invitrogen, Waltham, MA). The SMRTbell Express Template Prep Kit 2.0 (Pacific Biosciences, Menlo Park, CA) was used to prepare the genomic library for PacBio sequencing with BluePippin (Sage Science, Beverly, MA) size selection to target a library size of >7 kb. Genomic sequencing was performed on the PacBio Sequel II (Pacific Biosciences).

Sequencing yielded 317,377 PacBio reads totaling 300,011,149 bp (200x coverage), and their quality was examined using FastQC v.0.11.9.^1^ The PacBio reads were assembled with Canu v.2.1.1 (Koren et al., 2017) using the “pacbio-hifi” option, and the assembly produced five contigs with an N50 value of 1,528,390 bases and a GC content of 28.04%. Circlator (Hunt et al., 2015) was used to trim and circularize the contigs, which were further polished with Pilon, v.1.20 (Walker et al., 2014), eventually resulting in one chromosome when possible. The genome sequences were annotated using NCBI Prokaryotic Annotation Pipeline and RAST (Aziz et al., 2008). PlasmidFinder (Carattoli and Hasman, 2020) was used to identify plasmids in the genomes sequences.

### 2.3. Comparative genomic analysis

Genomes from other *Campylobacter* species were used to perform genome comparison with the four *C. hepaticus* genomes isolated in this study. 32 genomes of *Campylobacter* spp. were used including (seven genomes of *Campylobacter coli,* three *C. fetus,* eight *Campylobacter jejuni,* one *C. bilis*, and 13 other *C. hepaticus*). Information regarding strains, host, location, and accession numbers are detailed in Table 1.

**Table 1:** Source information for the genomes of *Campylobacter* sp. used in this study.

Accession number	Species	Strain	Host	Location	Year	References
NZ_WUED00000000.1	*C. bilis*	VicNov18	*Gallus gallus*	Victoria, Australia	2018	Phung et al. (2022)
NZ_CP083395.1	*C. coli*	C203018	*Gallus gallus*	Henan, China	2020	Direct submission
OU532556.1	*C. coli*	isolate 2	*Gallus gallus*	Sweden	2020	Direct submission
NZ_CP038868.1	*C. coli*	16SHKX65C	*Gallus gallus*	Shanghai, China	2016	Liu et al. (2020)
NZ_CP046317.1	*C. coli*	FDAARGOS_735	*Homo sapiens*	Virginia, United States	ND	Direct submission
NZ_CP076509.1	*C. coli*	R18.1828	*Homo sapiens*	Taiwan	2018	Liao et al. (2022)
CP066486.1	*C. coli*	PSU-31	*Homo sapiens*	Pennsylvania, United States	2011	Gunther et al. (2021)
NZ_CP059443.1	*C. fetus veneralis*	CFF00A031	*Bovine*	British Columbia, Canada	2000	Nadin-Davis et al. (2021)
NZ_CP072664.1	*C. fetus fetus*	CITCf01	*Homo sapiens*	Ireland	2017	Culligan et al. (2019)
NZ_CP043435.1	*C. fetus fetus*	NCTC 10354	*Homo sapiens*	United Kingdom	1952	Miller and Yee (2019)
NZ_CP031611.1	*C. hepaticus*	HV10	*Gallus gallus*	Victoria, Australia	2015	Van et al. (2019)
NZ_CP065357.1	*C. hepaticus*	UF2019SK1	*Gallus gallus*	Florida, United States	2019	Arukha et al. (2021)
CP104325	*C. hepaticus*	RBCL71delta	*Gallus gallus*	Georgia, United States	2021	This study
SAMN30753634	*C. hepaticus*	RBCL76delta	*Gallus gallus*	Georgia, United States	2021	This study
SAMN30753635	*C. hepaticus*	RBCL81delta	*Gallus gallus*	Georgia, United States	2021	This study
SAMN30753636	*C. hepaticus*	RBCL91delta	*Gallus gallus*	Georgia, United States	2021	This study
CP063536.1	*C. hepaticus*	USA52	*Gallus gallus*	Nebraska, United States	2019	Wu et al. (2021)
ERS1508458	*C. hepaticus*	S10-0209	*Gallus gallus*	England	2010	Crawshaw et al. (2015)
ERS1508462	*C. hepaticus*	S11-010	*Gallus gallus*	England	2011	Crawshaw et al. (2015)
ERS1508459	*C. hepaticus*	S11-0036	*Gallus gallus*	England	2011	Petrovska et al. (2017b)
ERS1508463	*C. hepaticus*	S11-0038	*Gallus gallus*	England	2011	Crawshaw et al. (2015)
ERS1508460	*C. hepaticus*	S11-0069	*Gallus gallus*	England	2011	Crawshaw et al. (2015)
ERS1508461	*C. hepaticus*	S11-0071	*Gallus gallus*	England	2011	Crawshaw et al. (2015)
ERS1508464	*C. hepaticus*	S11-5013	*Gallus gallus*	England	2011	Crawshaw et al. (2015)
ERS1508465	*C. hepaticus*	S12-002	*Gallus gallus*	England	2012	Crawshaw et al. (2015)
ERS1508466	*C. hepaticus*	S12-0322	*Gallus gallus*	England	2012	Crawshaw et al. (2015)
ERS1508467	*C. hepaticus*	S12-1018	*Gallus gallus*	England	2012	Crawshaw et al. (2015)
NZ_CP058299.1	*C. jejuni*	A9a	*Gallus gallus*	Kansas, United States	2003	Direct submission
NZ_CP023866.1	*C. jejuni*	FDAARGOS_421	*Gallus gallus*	Virginia, United States	1997	Direct submission
NZ_CP014744.1	*C. jejuni*	OD267	*Gallus gallus*	Oklahoma, United States	2009	Marasini and Fakhr (2016)
AL111168.1	*C. jejuni*	NCTC11168	*Homo sapiens*	United Kingdom	2000	Gundogdu et al. (2007)
NZ_CP071587.1	*C. jejuni*	RM1477	*Homo sapiens*	Florida, United States	1983	Heikema et al. (2021)
NZ_CP073712.1	*C. jejuni*	G1	*Homo sapiens*	United Kingdom	2014	Karlyshev (2021)
NZ_CP014344.1	*C. jejuni*	RM3194	*Homo sapiens*	Cape Town, South Africa	1994	Gunther et al. (2016)

The virulence genes were identified by searching directly against Virulence Factor Database 2022 using VFanalyzer (Liu et al., 2022), as well as using the ABRicate v.1.0.1,^2^ and SEED v.2.0 (Overbeek et al., 2005), available within RAST pipeline. On the ABRicate analysis, the presence of a gene was considered only with >90 and > 80% of coverage and identity, respectively. Antimicrobial resistance (AMR) genes and metal-resistance genes (MRG) were also identified through SEED, which classified the ARG and MRG in the sub-system “Resistance to antibiotic and toxic compounds.” In addition, MEGARes v.3.0 (Bonin et al., 2023) and ResFinder v.4.0 (Bortolaia et al., 2020) were also used to identify the AMR genes. Genome visualization and comparison were performed with GView.^3^

### 2.4. Pangenome and phylogenetic analysis

For the pangenome analysis, all four *C. hepaticus* genomes were annotated with Prokka v.1.14.6 (Seemann, 2014), and the output files were submitted to Roary v.3.13.0 (Page et al., 2015), which provides information about the core and accessory genes. A phylogenetic analysis based on the concatenated alignment of SNPs from all *Campylobacter* genomes mentioned above (Table 1) was performed with CSI Phylogeny v.1.4 (Kaas et al., 2014). CSI Phylogeny uses Burrows-Wheeles Aligner (BWA), SAMtools, and BEDtools to align nucleotide sequences followed by SNP calling, MUMmer to assess genome similarities, and FastTree 2 for maximum likelihood tree estimation (Ahrenfeldt et al., 2017). All default parameters of the software were applied. The final phylogenetic tree was created with the Interactive Tree of Life (iTOL) v.6.5.8 (Letunic and Bork, 2021).

### 2.5. Pangenome wide-association analysis (pan-GWAS)

An additional pangenome analysis for *Campylobacter* was performed to evaluate the association of genes with each *Campylobacter* species. All 34 *Campylobacter* genomes mentioned above (Table 1) were annotated with Prokka v.1.14.6 (Seemann, 2014), and the output files were submitted to Roary v.3.13.0 (Page et al., 2015). The gene presence or absence table generated by Roary was used as input file in Scoary v.1.6.16 (Brynildsrud et al., 2016), with a *p*-value cut-off of 0.05, adjusted via the Bonferroni correction, to establish gene clusters associated with each trait (*Campylobacter* sp.).

### 2.6. Statistical analysis

Statistical analysis regarding the genetic differences between *C. hepaticus* and other *Campylobacter* species were performed using GraphPad Prism 9. The Kruskal-Wallis test was performed to compare each subsystem parameter between the *Campylobacter* species.

## 3. Results

### 3.1. Characterization of *Campylobacter hepaticus* genomes from Georgia, United States

One *C. hepaticus* genome was closed into a chromosome (RBCL71delta), and the other three genomes were assembled into contigs (Becerra et al., 2023a). The genome from *C. hepaticus* RBCL71delta has a GC content of 28.03% and consists of 1,516,079 base pairs. It contains 1,488 predicted protein-coding sequences (CDS) encoding 54 RNAs (9 rRNA genes and 42 tRNAs and 3ncRNAs). The other three *C. hepaticus* genomes have an average GC content and size of 28.05% and 1,558,574 base pairs, respectively (Table 2). No plasmids were identified in any of the sequenced *C. hepaticus* genomes.

**Table 2:** Genome information for the *C. hepaticus* isolates from Georgia, United States.

Species	Strain	Host	Source	N50	Contigs	GC content	Size (bp)	Accession number
*C. hepaticus*	RBCL71delta	*Gallus gallus*	Bile	1,516,079	1	28.03%	1,516,079	CP104325
*C. hepaticus*	RBCL76delta	*Gallus gallus*	Bile	1,516,087	4	28.07%	1,560,236	JAOBQM000000000
*C. hepaticus*	RBCL81delta	*Gallus gallus*	Bile	1,516,087	3	28.06%	1,564,828	JAOBQL000000000
*C. hepaticus*	RBCL91delta	*Gallus gallus*	Bile	1,487,452	2	28.04%	1,550,659	JAOBQK000000000

**Figure 1:**
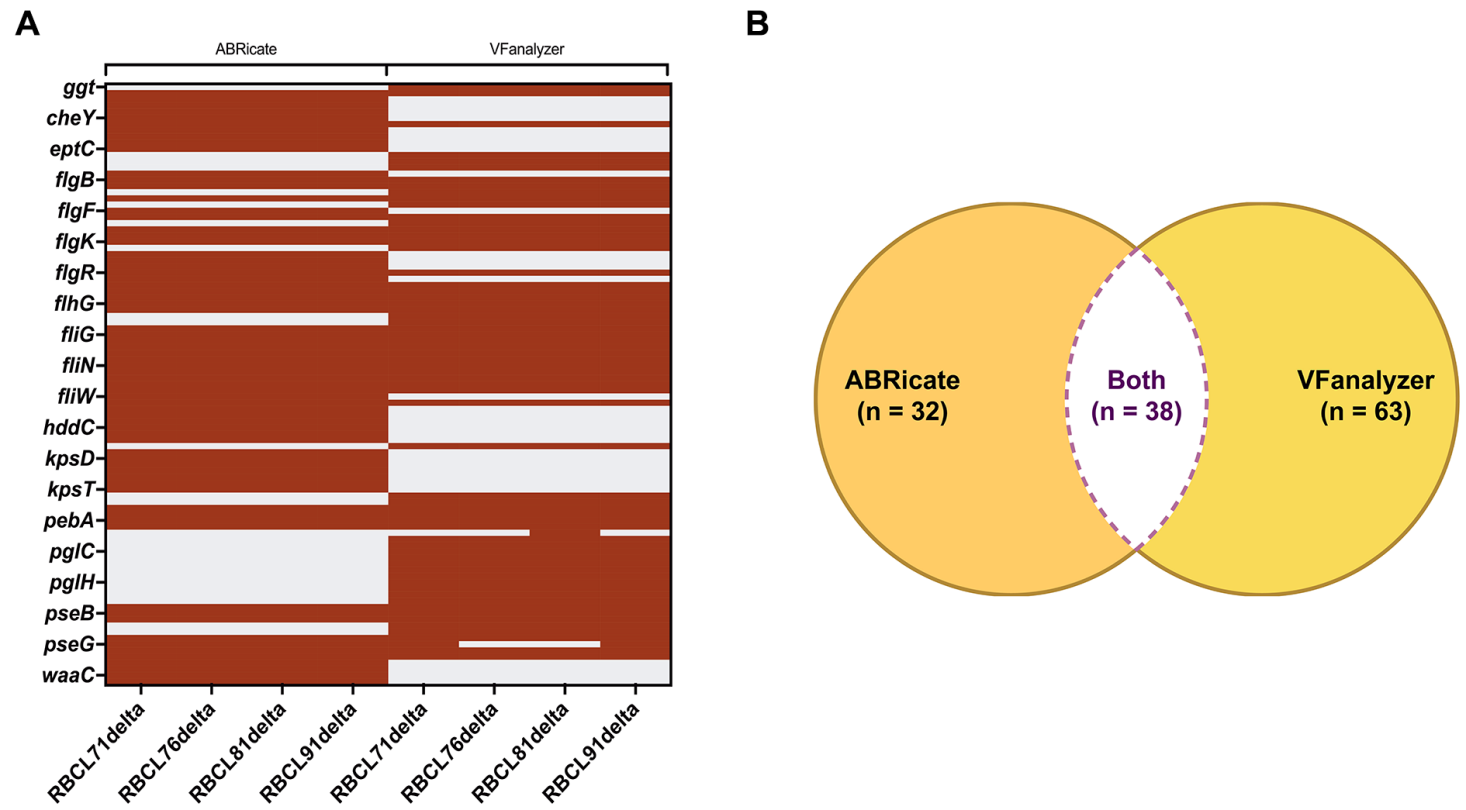
Comparison of the results provided by ABRicate and VFanalyzer. **(A)** Heatmap with the genes identified by each database. The gene identification is represented by maroon stripes, and the non-identification is represented by gray stripes. **(B)** Using both databases, a total of 133 genes were identified in the four *C. hepaticus* genomes, and only 38 genes were identified by both databases.

Using the virulence factor database (VFDB) from ABRicate, 70 virulence genes were identified in the four sequenced genomes. The VFanalyzer from Virulence Factor Database website had predicted 101 virulence genes in the four sequenced genomes, of which an average of 35 were unidentified, classified by the same tool only by their predicted function (Figure 1). Most of those genes are related to adherence, colonization, immune evasion, motility, and glycosylation. The genes identified through both methods are included in Supplementary Table S1. Only 38 genes were identified by both ABRicate and VFanalyzer. Use of two search tools gave better results than a single search tool alone.

The *Campylobacter* invasion antigen B (*ciaB*) was identified in all four *C. hepaticus* isolates, confirming that the isolates belong to this *Campylobacter* species, since this gene is an essential virulence factor for *Campylobacter* that plays a key role in the invasion of *C. jejuni, C. coli, Campylobacter lari, and Campylobacter rectus* into human intestinal cells (Konkel et al., 1999; Fouts et al., 2005; Onozato et al., 2009; Lagier and Threadgill, 2014) and *ciaB* functions as a target of the type III secretion system (Konkel et al., 1999). In the present study, the *ciaB* gene identified in the four *C. hepaticus* RBCL strains was submitted to BLAST and showed a match only with the other three *C. hepaticus* genomes in the NCBI database (*C. hepaticus* HV10, *C. hepaticus* UF2019SK1, and *C. hepaticus* USA52) with an identity and query cover of 100%.

Using the SEED Viewer from RAST Annotations, five genes related to bacteriocin production were identified, including the *cvpA* gene that encodes the colicin V production protein (Table 3). Genes related to multidrug efflux pumps were also identified, including the *cmeABC* operon, a system that has been extensively studied in *C. jejuni* species (Lin et al., 2002, 2003). The four sequenced genomes also harbored genes that promote resistance to copper, cobalt, zinc, and cadmium, and fluoroquinolones.

**Table 3:** Relevant virulence genes identified in *Campylobacter hepaticus* RBCL71delta using RAST and virulence factor database.

Species	Subsystem	Description	Gene	Start	Stop	Length (pb)	CF1	CF2	CF3
*Campylobacter hepaticus* RBCL71delta*									
	Invasion								
			*ciaB*	634,245	636,080	1836	+	+	+
	Multidrug efflux pump								
		CmeABC multidrug efflux system	*cmeA*	218,982	217,879	1,103	+	+	+
		CmeABC multidrug efflux system	*cmeB*	214,757	217,879	3,123	+	+	+
		CmeABC multidrug efflux system	*cmeC*	214,764	213,325	1,439	+	+	+
		CmeABC multidrug efflux system repressor	*cmeR*	219,731	219,093	638	−	−	+
		MATE family efflux transporter	N/D	386,639	387,955	1,316	+	+	+
		TolC family protein (outer membrane efflux protein)	*tolC*	736,147	737,430	1,283	−	−	−
		efflux RND transporter periplasmic adaptor subunit	N/D	737,427	738,167	740	−	−	−
		efflux RND transporter permease subunit	*acrB*	738,210	741,188	2,978	−	−	−
	Resistance to fluoroquinolones								
		DNA gyrase A	*gyrA*	732,884	730,284	2,601	+	+	+
		DNA gyrase B	*gyrB*	2,573	4,882	2,309	+	+	+
	Resistance to heavy metals								
		Magnesium and cobalt transport protein	N/D	473,267	472,299	969	+	+	+
		CopG protein – copper homeostasis	*copG*	38,111	38,554	443	+	+	+
		cobalt-zinc-cadmium resistance	N/D	672,672	671,785	888	+	+	+
		Copper homeostasis and tolerance	*cutE*	794,054	795,175	1,122	+	+	+
		Heavy metal translocation P-type ATPase	*zntA*	860,168	857,817	2,352	+	+	+
				865,803	863,698	2,106	+	+	+
		Multicopper oxidase	*cueO*	1,250,462	1,248,933	1,530	+	+	+
		Heavy metal associated domain	N/D	1,276,002	1,276,661	660	+	+	+
		Multidrug resistance transporter, Bcr/CflA family	N/D	1,388,967	1,387,762	1,205	−	−	−
		Magnesium and cobalt efflux protein	N/D	1,499,625	1,500,911	1,287	+	+	+
	Bacteriocins								
		colicin V production	*cvpA*	245,976	246,533	557	+	+	+
		tRNA pseudouridine synthase A	*truA*	562,338	563,060	722	+	+	+
		Dihydrofolate synthase / Folylpolyglutamate synthase	N/D	786,750	785,587	1,163	+	+	+
		Acetyl-coenzyme A carboxyl transferase beta chain	*accD*	1,461,104	1,460,262	842	+	+	+
		Amidophosphoribosyltransferase	*purF*	1,511,192	1,509,855	1,337	+	+	+

*All genes identified in RBCL71delta were also identified in other RBCL strains. CF1, *C. fetus* CITCf01; CF2, *C. fetus* NCTC 10354; CF3, *C. fetus* CFF00A031. N/D: non determined.

### 3.2. Comparative genomic analysis and pan-genome

A comparison of the genome sequences of the 17 *C. hepaticus* genomes showed a high conservation profile with an average identity of 99.91% against the reference genome, *C. hepaticus* HV10 (Supplementary Table S2). Pan-genome analysis identified 2,102 genes classified into the core, shell, and cloud genes (Supplementary Figures S1, S2). The *C. hepaticus* core was composed of 1,328 genes, while 366 and 408 genes were considered as shell and cloud genes, respectively.

**Figure 2:**
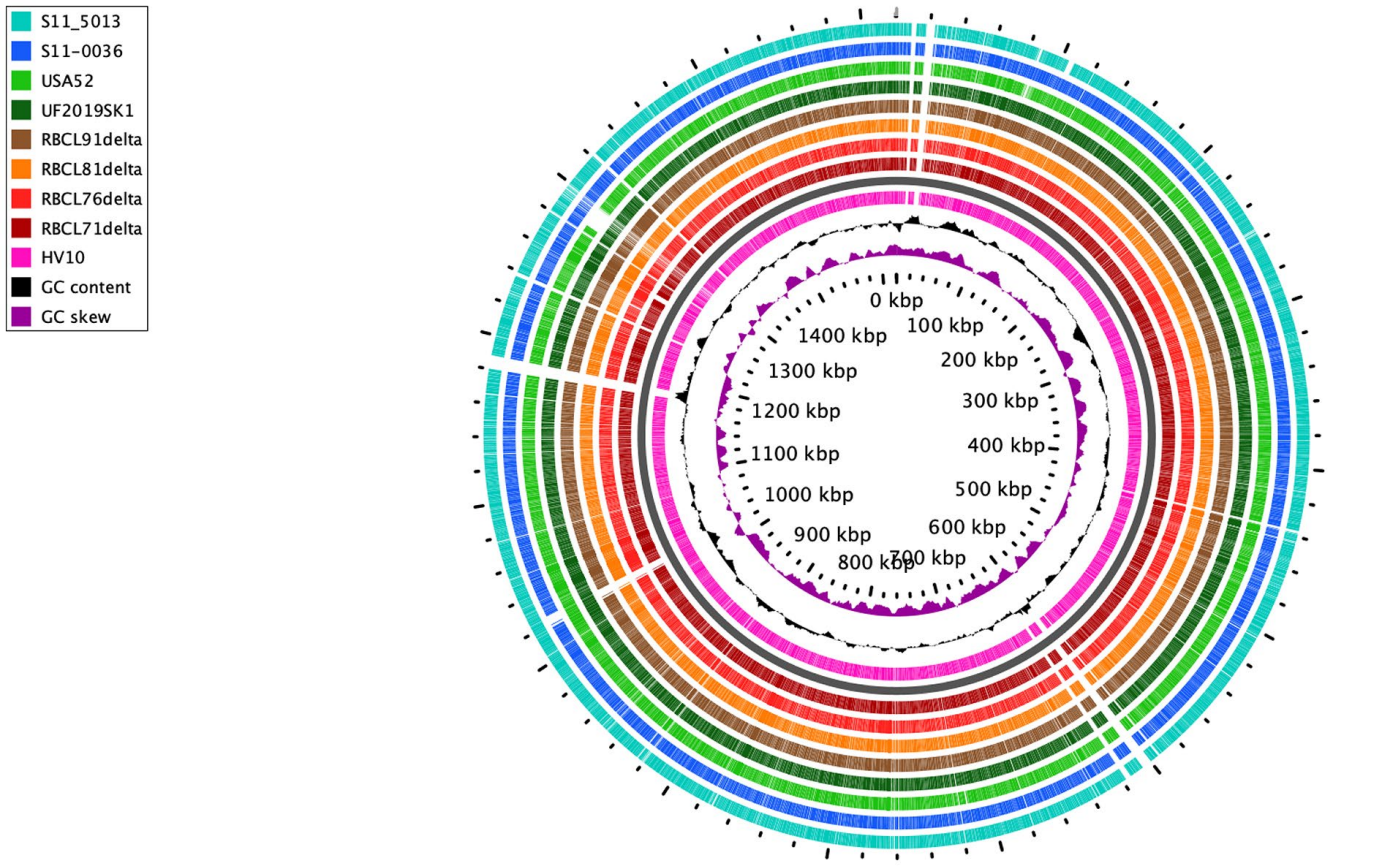
Circular visualization of 9 *C. hepaticus* genomes from different countries. United States: RBCL71delta, RBCL76delta, RBCL81delta, RBCL91delta, UF2019SK1, and USA52; United Kingdom: S11-5013 and S11-0036; Australia: HV10 (reference genome). The gray ring is the backbone to separate the reference genome (pink ring) from the other genomes.

For better comprehension, circular comparison visualization of 9 representative *C. hepaticus* complete genomes was performed based on a BLAST analysis (Figure 2), followed by a pan genome analysis (Supplementary Figure S3), including the reference genome, *C. hepaticus* HV10. This analysis found that there were many conserved regions between the 9 representative *C. hepaticus* complete genomes when compared, including some identical gaps. However, next to the 1,000 kbp region, the four genomes sequenced in this study showed a distinct gap compared to the reference genome. This same gap was also observed in the *C. hepaticus* S11-0036, from the United Kingdom. The *C. hepaticus* USA52 also has an exclusive gap next to the 1,300 kbp region, observed only in this genome, likely due to some gene deletion. This gap is associated with 18 genes identified in all other *C. hepaticus* genomes, most were codified as non-identified proteins, which were classified as hypothetical proteins in the annotated genomes. However, in this region, some genes codified to the LexA family transcriptional regulator, the type II toxin-antitoxin system RelE/ParE family toxin, and the helix-turn-helix domain-containing protein, which are not present in the *C. hepaticus* USA52 isolate (Supplementary Figure S4).

**Figure 3:**
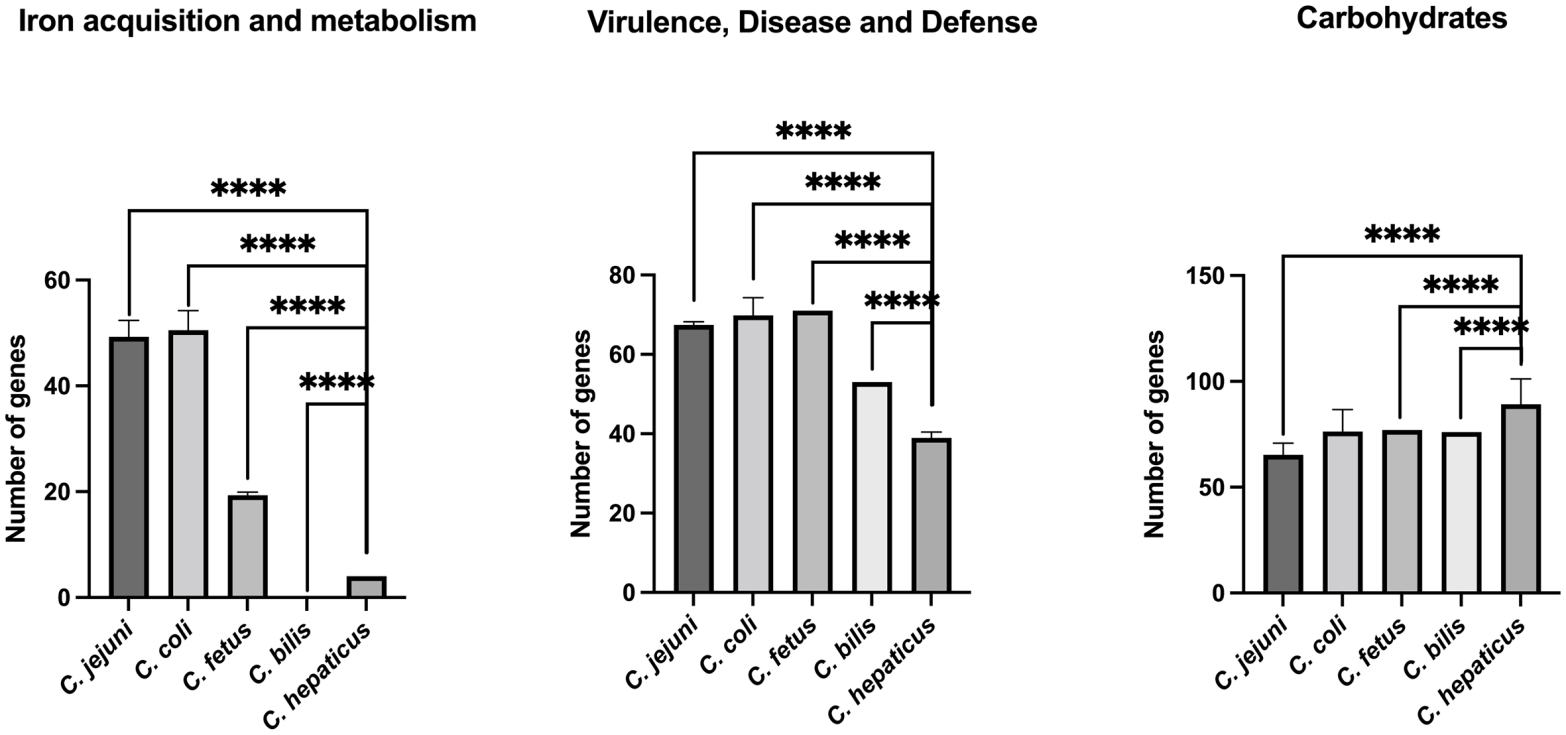
Comparison of *C. hepaticus* with *C. jejuni, C. coli, C. fetus,* and *C. bilis* genomes using sub-systems classification from RAST. **** indicates a *p* value <0.0001. *C. hepaticus* isolates: HV10, UF2019SK1, USA52, S10-0209, S11-010, S11-0036, S11-0038, S11-0069, S11-0071, S11-5013, S12-002, S12-0322, S12-1018, RBCL71delta, RBCL76delta, RBCL81delta, RBCL91delta. *C. jejuni* isolates: NCTC11168, A9a, FDAARGOS_421, OD267, RM1477, G1, RM3194. *C. coli* isolates: C203018, isolate2, 16SHKX65C, FDAARGOS_735, R18.1828, PSU-31. *C. fetus* isolates: NCTC 10354, CFF00A031, CITCf01. *C. bilis* isolate: VicNov18.

Using the classification of sub-systems from RAST, a comparative analysis of virulence factors between different *Campylobacter* species was performed. All 34 *Campylobacter* genomes were included in this analysis (See Methods section). It was found that compared with other *Campylobacter* species examined (*C. jejuni, C. coli, C. fetus,* and *C. bilis*), *C. hepaticus* has a significantly lower number of genes classified in the following functional groups: iron acquisition, virulence, disease, and defense (Figure 3). In contrast, the *C. hepaticus* genomes harbor a significantly greater number of genes related to carbohydrate metabolism than other *Campylobacter* species, except for *C. coli*. It was also found that in contrast to all other *Campylobacter* genomes, the *C. bilis* VicNov18 genome does not appear to harbor any genes related to the iron acquisition system (Figure 3).

The pangenome analysis using all *Campylobacter* genomes identified a total of 15.145 genes, classified into soft, shell, and cloud genes. No gene was identified in more than 99% of the isolates to be considered as core genes. The pan-WGAS revealed that a total of 73, 50, and 16 genes were associated with *C. hepaticus, C. jejuni,* and *C. coli*, respectively (*p* < 0.05; Supplementary Table S3). However, the analysis did not reveal any significant associations between *C. bilis* and *C. fetus* with specific genes. Although some virulence genes were associated to *C. hepticus,* such as *flgE*, *flaA,* and *flab*, most of the genes associated were hypothetical proteins, and further analysis linking the Scoary results and this pathogen is required.

### 3.3. Phylogenetic analysis

**Figure 4:**
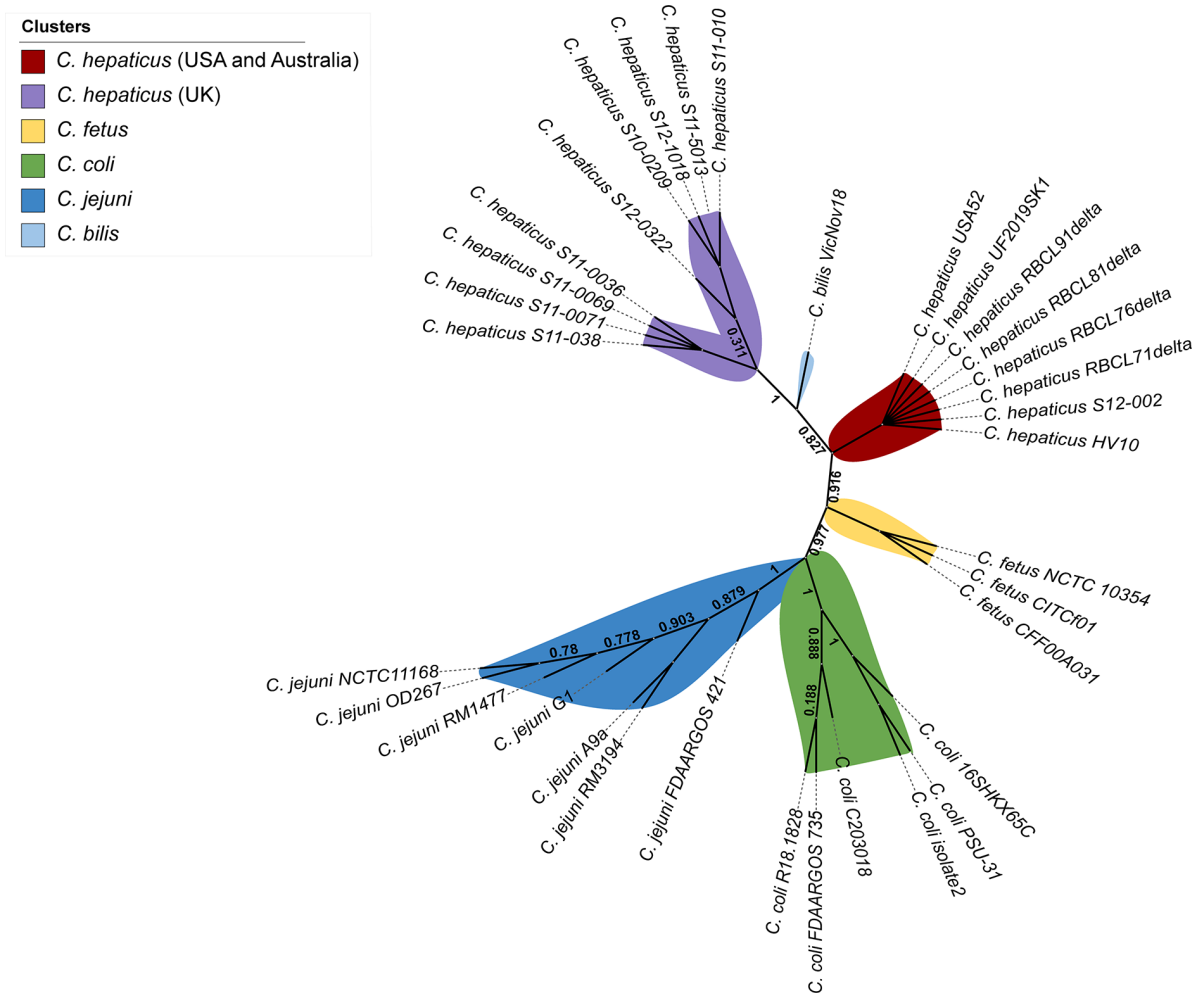
Whole genome unrooted phylogenetic tree from all 34 *Campylobacter* genomes inferred by CSI Phylogeny based on the concatenated alignment of SNPs.

Single nucleotide polymorphisms (SNPs) were identified from alignments of the examined *Campylobacter* whole-genomes. Figure 4 shows that *C. hepaticus* genomes were grouped into two distinct groups, and *C. bilis* VicNov18 was located as an intermediate group between them. One cluster consists of 9 *C. hepaticus* genomes from the UK, while the second cluster includes all other *C. hepaticus* genomes from the United States and Australia. However, isolate S12-002 from the UK was not grouped in the UK cluster since it had ~1–3 SNPs differences from the UK genomes and no SNPs differences in comparison with the United States and Australia genomes (Supplementary Figure S5). The highest number of SNPs observed among the *C. hepaticus* genomes was 4, indicating that this species has a well-conserved genome (Supplementary Figure S5). It was also found that the *C. fetus* genomes (*C. fetus* subsp. *fetus* and *C. fetus* subsp. *veneralis*) are phylogenetically closer to *C. hepaticus* (~16–19 SNPs) than *C. coli* and *C. jejuni*. High bootstrapping values support these data.

## 4. Discussion

### 4.1. *Campylobacter hepaticus* genome analysis

This study found that the four genomes characterized (RBCL 71, 76, 81 and 91 delta) differed slightly from the type strain *C. hepaticus* HV10. Using the closed genome RBCL71delta for comparison it has a genome size of 1,516,079 bp compared to 1,520,669 bp for HV10, this is also reflected in a slightly lower GC content (28.03%) (compared to 28.2% for HV10) (Van et al., 2019). However, a few more coding sequences were detected in the RBCL71 delta genome (1,488) compared to *C. hepaticus* HV10 (1,474). The four sequenced genomes are slightly larger than the USA52 (Wu et al., 2021) and similar in size to UF2019SK (Arukha et al., 2021), the only two other US *C. hepaticus* genomes available.

Of interest, no plasmids were detected in any of the four sequenced genomes analyzed in this study or the other two US genomes (Arukha et al., 2021; Wu et al., 2021), while Van and colleagues (Van et al., 2019) only detected plasmids in five of 14 *C. hepaticus* isolates from Australia that were analyzed, suggesting that mobile elements such as plasmids may not be a common trait of *C. hepaticus*.

The four new *C. hepaticus* genomes sequenced in this study would appear to be relatively conserved with a match level of 99.95% with *C. hepaticus* HV10. This match is greater than that reported by Van and collaborators (Van et al., 2019) where comparison of HV10 with a collection of 22 *C. hepaticus* genomes consisting of 13 Australian genomes and 9 UK genomes found an identity match of 95.19%. The four genomes sequenced in this study are a first report of *C. hepaticus* in US pasture raised poultry.

In this study, phylogenetic analysis was performed based on the concatenated alignment of SNPs, and this analysis provides a SNP matrix from the whole-genome alignment. A low number of SNPs was detected among the *C. hepaticus* genomes, but level present might be enough to initiate an evolutionary process that distinguishes the UK genomes from the *C. hepaticus* genomes from the United States and Australia. Moreover, the lowest number of SNPs among the *C. hepaticus* genomes indicates that this species has a conserved genome aside from geographical differences. In addition, considering that *C. hepaticus* was described in 2016 (Van et al., 2016) and just a few genomes are currently available, the lower number of SNPs is likely plausible. The four *C. hepaticus* genomes from Georgia (United States) showed 99.95% sequence identity compared to the reference genome *C. hepaticus* HV10, which sustains the hypothesis that these genomes are highly conserved.

### 4.2. Virulence genes analysis and comparison of databases

Virulence gene analysis of the four genomes using two different search databases found significant differences in the number of genes identified as virulence genes. The database ABRicate identified 70 virulence genes among the four sequenced *C. hepaticus* genomes compared to 101 using the VFanalyzer directly from Virulence Factor Database (VFDB) website. When both search engines were compared, only 38 of the identified virulence genes were common to both search tools. Of interest, even with regular updates, the ABRicate database has the same number of sequences since 2019, when the software was curated with a database from 2016 (Chen et al., 2016), while the newer search database VFanalyzer appears more current (Liu et al., 2022) and was updated in 2022. This suggests that the VFDB online resource is more accurate in finding virulence genes if compared by the number of genes found, however use of both search tools resulted in a better overall outcome in identifying all virulence genes in the four genomes examined. This study highlights the importance of using more than one analysis method to evaluate whole genome sequences, since their different approaches alone could provide false-negative results. To our knowledge, this is the first time that two different search databases were compared for output in terms of virulence genes identified for *C. hepaticus*.

Among the virulence genes identified, the *ciaB* gene, associated with *Campylobacter* invasion was found in all four genomes and showed 100% identity match to *C. hepaticus* HV10, UF2019SK1, and USA52. Similar results were identified by Onozato et al. (2009) when compared the nucleotide sequence of *ciaB* gene from *C. lari* isolates with the sequences from *C. coli* and *C. jejuni* isolates. The authors suggested that the nucleotide sequence information of the *ciaB* gene has molecular discrimination efficacy among the *Campylobacter* species analyzed (Onozato et al., 2009). This finding raises the hypothesis that CiaB might be a well conserved gene among *C. hepaticus* species and further studies are required to identify its potential as a target for antimicrobial or diagnostics tools.

### 4.3. Multi drug efflux pump CmeABC and virulence factors of *Campylobacter hepaticus*

Although no plasmid or mobile elements were detected in our *C. hepaticus* genomes, some genes first characterized in *C. jejuni* and *C. coli* were identified in the *C. hepaticus* RBCL genomes. The multidrug efflux pump CmeABC was identified in all four genomes and has also been described by Van and colleagues (Van et al., 2019) in *C. hepaticus* HV10. This operon is also present in the *C. hepaticus* strains USA52 and UF2019SK1, from Iowa and Florida, respectively. CmeABC plays an active role in the intrinsic resistance of *C. jejuni* (Lin et al., 2002), has been extensively characterized in *C. jejuni* (Lin et al., 2002, 2003; Lin et al., 2005a,b) and has also been detected in *C. coli* and other *Campylobacter* species (Olah et al., 2006; Guo et al., 2010) appearing to have a similar genomic organization in all species. Functional analysis identified CmeABC has a role in the efflux of drugs and other antimicrobial agents out of the bacterial host and contributes to the colonization of the poultry host (Lin et al., 2003). Lin and colleagues (Lin et al., 2005b) demonstrated that in the presence of bile salts, there was elevated expression of *cmeABC* genes in *C. jejuni* and increased resistance to multiple antimicrobials. These studies also found bile resistance was a natural function of CmeABC. In the present study, this operon was observed in all *Campylobacter* species, as well as its repressor gene, *cmeR*. *C. hepaticus* and *C. bilis* have been detected and recovered from bile and liver samples of birds affected with SLD (Van T. T. H. et al., 2017; Phung et al., 2022; Van et al., 2022). The presence of CmeABC in *C. hepaticus* and *C. bilis* might be an essential factor in these pathogens surviving and colonizing the liver and gall bladder of birds.

In the current study, detection of a full CmeABC operon in *C. hepaticus* likely plays a key role in the survival and host adaptation of *C. hepaticus* to the chicken host liver and gall bladder. All four *C. hepaticus* strains sequenced were isolated from bile samples (Becerra et al., 2023b) and *C. hepaticus* has been detected in the liver of infected birds with or without characteristic spots using PCR analysis (Becerra et al., 2023b). Given that the CmeABC operon functions to promote resistance to bile then *C. hepaticus* would appear to have acquired the ability to survive and flourish in this host niche environment.

In concert with CmeABC, genomic analysis also identified an overall reduction in the size of the *C. hepaticus* genome compared to other members of the *Campylobacteriaceae*. Of note, reductions were observed in the number of genes associated with the iron acquisition systems (Figure 3). A similar observation was noted for *C. hepaticus* HV10 (Van et al., 2019). Petrovska and colleagues (Petrovska et al., 2017a,b) also noted a genome reduction in *C. hepaticus* genomes when analyzed for their association with the poultry host. This loss of system function may be related to the niche environment occupied by *C. hepaticus* which is the poultry liver and ease of access to specific factors. The liver itself functions to maintain systemic iron balance for the host and also acts as a storage site for excess iron. Therefore this niche may be the perfect environment for *C. hepaticus* and working in concert with CmeABC may serve as mechanisms to promote and maintain the *C. hepaticus* population in the liver/bile of infected birds, an observation also supported by Van and colleagues (Van et al., 2019). Further work to validate this observation is required, however, recent work using an iron depletion assay demonstrated the inability of *C. hepaticus* to grow in the absence of available free iron supporting the observation that the iron metabolism pathways become eliminated when the metabolites are readily available (Petrovska et al., 2017a,b). Also of note, when the *C. bilis* genome was included in the current analysis it also failed to harbor any iron acquisition systems supporting its role as another *C. hepaticus*-like strain with a specific niche in the poultry host.

When all four genomes were examined for other metabolism systems, genes associated with carbohydrate metabolism systems were found to have a significantly greater prevalence in *C. hepaticus* compared to all other members of the genus (*C. jejuni*, *C. fetus* and *C. bilis*) except *C. coli*. Carbohydrate systems function as pathways that the organism can use to function in a carbohydrate rich environment such as the liver (Petrovska et al., 2017a,b; Van et al., 2019). Generally, these systems are not as common in the genus *Campylobacter* as the organism is characterized as non-glycolytic (Van et al., 2019) but the presence of greater numbers of genes linked with carbohydrate metabolism may be necessary to cope in a carbohydrate rich environment such as the liver (Petrovska et al., 2017a,b).

### 4.4. Metal and drug resistance genes

Although none of our four sequenced genomes were found to harbor plasmids or the *tetO* gene which is often found associated with *Campylobacter* species, the four sequenced strains did, however, show the presence of genes associated with resistance to heavy metals including copper, cobalt, zinc and cadmium and the presence of genes linked with fluoroquinolone resistance (Table 2). Additionally, no genes related to tetracycline or any other antimicrobial class were found in the four *C. hepaticus* genomes.

One potential reason for the lack of *tetO* and other antimicrobial resistance genes among the four sequenced isolates could be related to the farm source: all strains originated on an organic farm where no antibiotics are used and if this farm has been in operation for a number of years there may be a lack of persistence of *tetO* in the absence of a selective pressure. However, some researchers suggest that resistance can still persist in the absence of selective pressure. In the case of the current farm however, little information is available as to the age of the farm and length of time operational as an organic producer but it has been estimated to be more than 5 years, and any comments on resistance persistence would be speculation.

### 4.5. Lineage effects on SNP analysis

The present study is the first to perform a whole genome comparison among *C. hepaticus* genomes. The comparison between *C. hepaticus* and other *Campylobacter* sp. genomes showed that the lowest number of SNPs was observed between *C. hepaticus* and *C. fetus* genomes. This close phylogenetic relationship was never identified before, probably because previous studies examining *C. hepaticus* phylogeny failed to include *C. fetus* in genomes their analysis. Some studies included *C. fetus* in their phylogenetic analysis (Petrovska et al., 2017b; Gregory et al., 2018); however, their analyses were based on 16S rRNA genes and rMLST, respectively, instead of the whole genome. In the current study, *C. fetus* was included because of its ability to colonize humans and livestock, and is a potential zoonotic pathogen (Chala et al., 2021). Iraola and colleagues (Iraola et al., 2017) demonstrated that *C. fetus* may have originated as a human pathobiont around 10,500 years ago and may have “jumped” into cattle and adapted as a venereal and abortive pathogen during the livestock domestication period.

Compared with all other *Campylobacter* genomes, *C. hepaticus, C. bilis,* and *C. fetus* have the lowest number of genes associated with iron acquisition and metabolism, while *C. jejuni* and *C. coli* have a similar number of genes for this system. *C. fetus* infects the intestinal tract of several mammalian species and induces infertility and abortion in cattle and sheep due to its ability to colonize and translocate to the placenta (Grogono-Thomas et al., 2000; Iraola et al., 2016). Chemotaxis assays showed that in high concentrations, *C. fetus* is a chemoattracted to ferrous iron, pyruvate, fumarate, L-serine, L-aspartate, and L-glutamate, but hormones such as estradiol and progesterone are non-chemotactic for this species (Haas et al., 2021). The placenta is an environment rich in iron since this is essential for development of the fetus (Peter, 2013; Cao and Fleming, 2016), and its composition might support the translocation of *C. fetus* from the gastrointestinal tract to the placenta by chemotaxis. Since this environment is a free nutrient source, the bacteria do not need a complete apparatus for iron metabolism, which explains the lower number of genes related to iron acquisition and metabolism as observed in this study.

In addition, *C. fetus* also harbors genes associated to resistance to fluoroquinolones, copper homeostasis and tolerance, and cobalt-zinc-cadmium resistance (Table 2), genes that were also evident in *C. hepaticus*. All these similarities could help explain the close phylogenetic relationship between *C. hepaticus*, *C. bilis* and *C. fetus* species and their ability to hide out in exclusive niches of their animal host.

## 5. Conclusion

In conclusion, this study provides first evidence and analysis of *C. hepaticus* genomes from pasture raised birds implicated in disease in the US. Genomic analysis supports the close association between these genomes and the type strain *C. hepaticus* HV10. Novel findings of this study include the close phylogenetic relationship between the four sequenced *C. hepaticus* genomes and *C. fetus* suggesting that the host niche for these organisms leads to reduced functional genes required to allow survival and persistence. Despite its limitations, this study certainly adds to our understanding of the *C. hepaticus* phylogeny, pathogenicity, and developing control and prevention strategies for this emerging pathogen.

## Data availability statement

The original contributions presented in the study are included in the article/supplementary material and in online repositories. The names of the repository/repositories and accession number(s) can be found below: *C. hepaticus* RBCL71delta: GenBank, PRJNA878518; the other three genomes: GenBank, PRJNA878793; the raw reads: NCBI SRA, SRR21491726, SRR21504727, SRR21504726, and SRR21504725. Requests for strains may be made by contacting the corresponding author.

## Ethics statement

The animal study was reviewed and approved by UGA’s IACUC committee approval number A2020 02-007-Y3-A2.

## Author contributions

JI-L carried out the genomic analysis including genome sequence and assembly, comparative analysis, and drafted and edited the manuscript. RB carried out analysis of farm samples that generated the genomes and drafted and edited the manuscript. CL funded the study, isolated the strains that were used for genome sequencing, and drafted and edited the manuscript. All authors contributed to the article and approved the submitted version.

## Funding

Funding support for this project was made available through the Dean’s Office College of Veterinary Medicine, University of Georgia. The funders had no role in study design, collection of data, analysis or interpretation of data or in writing of the manuscript.

## Conflict of interest

The authors declare that the research was conducted in the absence of any commercial or financial relationships that could be construed as a potential conflict of interest.

## Publisher’s note

All claims expressed in this article are solely those of the authors and do not necessarily represent those of their affiliated organizations, or those of the publisher, the editors and the reviewers. Any product that may be evaluated in this article, or claim that may be made by its manufacturer, is not guaranteed or endorsed by the publisher.

## Footnotes

^1^ https://www.bioinformatics.babraham.ac.uk/projects/fastqc/

^2^ https://github.com/tseemann/abricate

^3^ https://server.gview.ca/

## Glossary

BLAST: Basic local alignment search tool
BWA: Burrows-Wheeler Aligner
CDS: Coding sequence
*ciaB*: *Campylobacter* invasion antigen gene
CmeABC: *Campylobacter* multidrug efflux pump
DNA: Deoxyribonucleic acid
GC: Guanine-cytosine
HMW: High molecular weight
iTOL: Interactive Tree of Life
kb: kilobases
kbp: kilobase pairs
MLST: Multilocus sequence typing
NCBI: National Center for Biotechnology Information
ncRNA: Non-coding ribonucleic acid
PacBio: Pacific Biosciences
PCR: Polymerase Chain Reaction
RAST: Rapid Annotation using Subsystem Technology
RNA: Ribonucleic Acid
rRNA: Ribosomal ribonucleic Acid
SEED: Subsystem-based annotation approach
SLD: Spotty liver disease
SNP: Single nucleotide polymorphisms
*tetO*: Tetracycline resistance gene
tRNA: Transfer ribonucleic acid
UK: United Kingdom
USA: United States
VFDB: Virulence Factor Database
