# Supplementary material for: Comparative genomic analysis of Campylobacter hepaticus genomes associated with spotty liver disease, Georgia, United States
Source: Front Microbiol. 2023 Jun 29;14:1215769. doi: 10.3389/fmicb.2023.1215769 (PMC10343453; doi:10.3389/fmicb.2023.1215769)
Supplement: Supplementary file 1 [file Data_Sheet_1.docx]

Supplementary Material

# Supplementary Figures


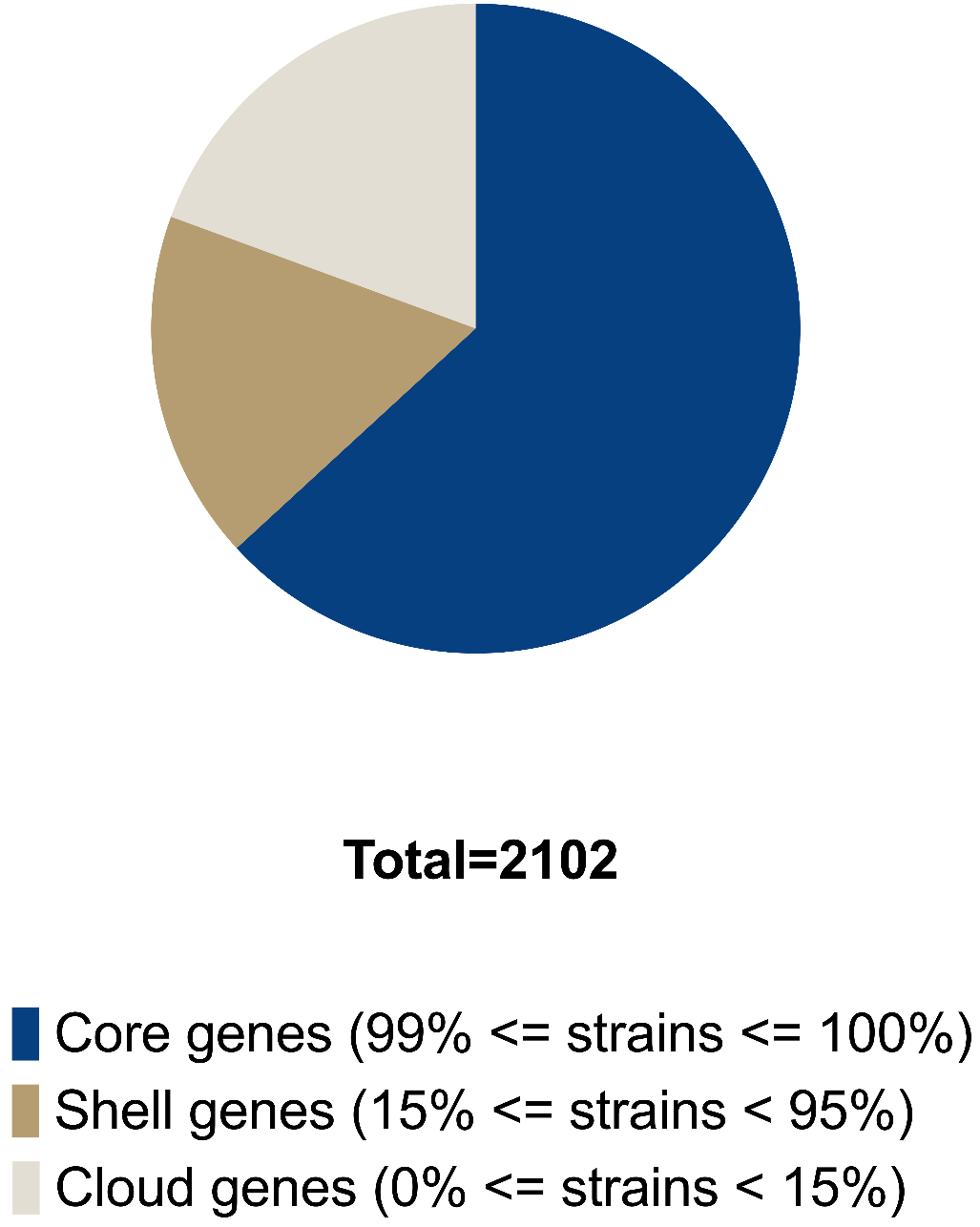


**Supplementary Figure S1.** Core and Pan Genome pie-chart.


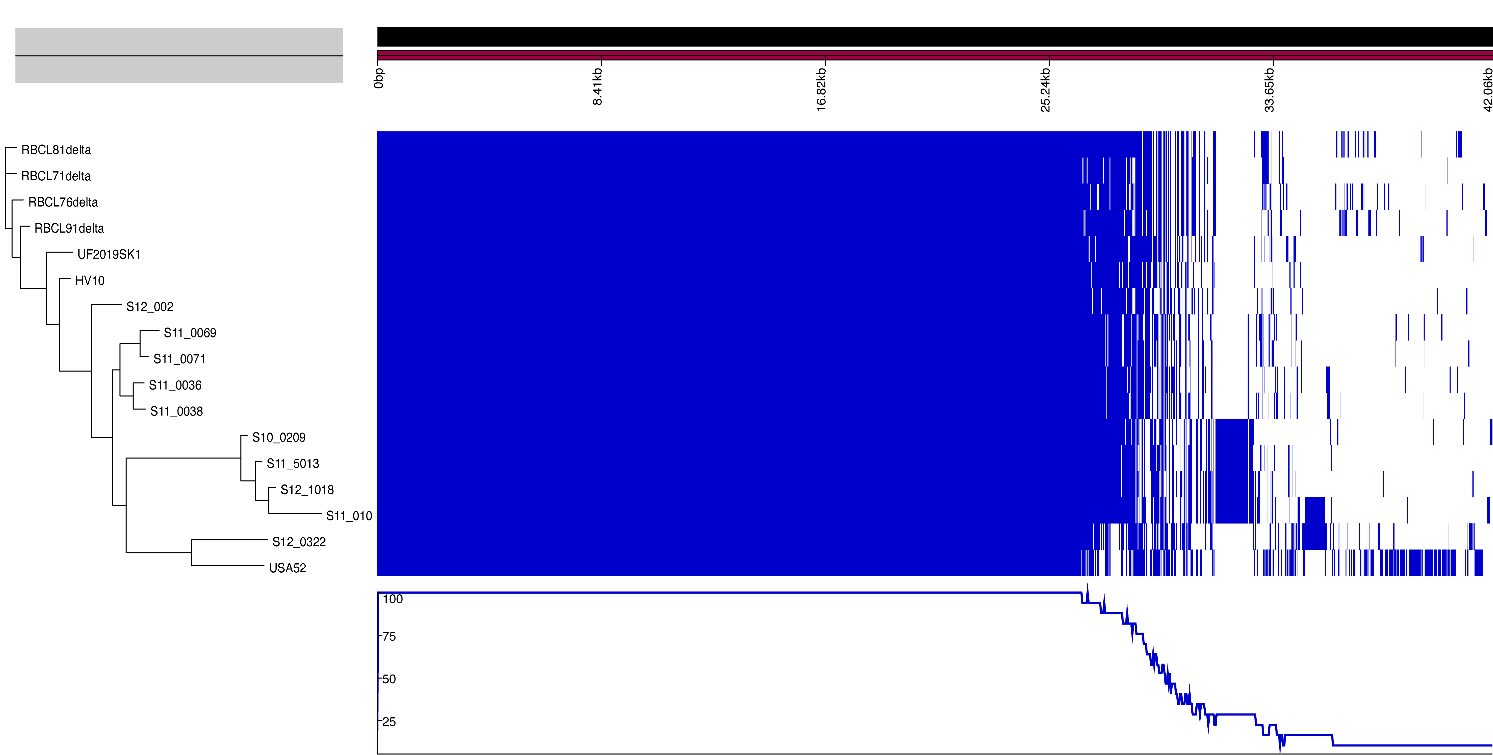


**Supplementary Figure S2.** Pan Genome phylogenetic tree and distribution of the genes.


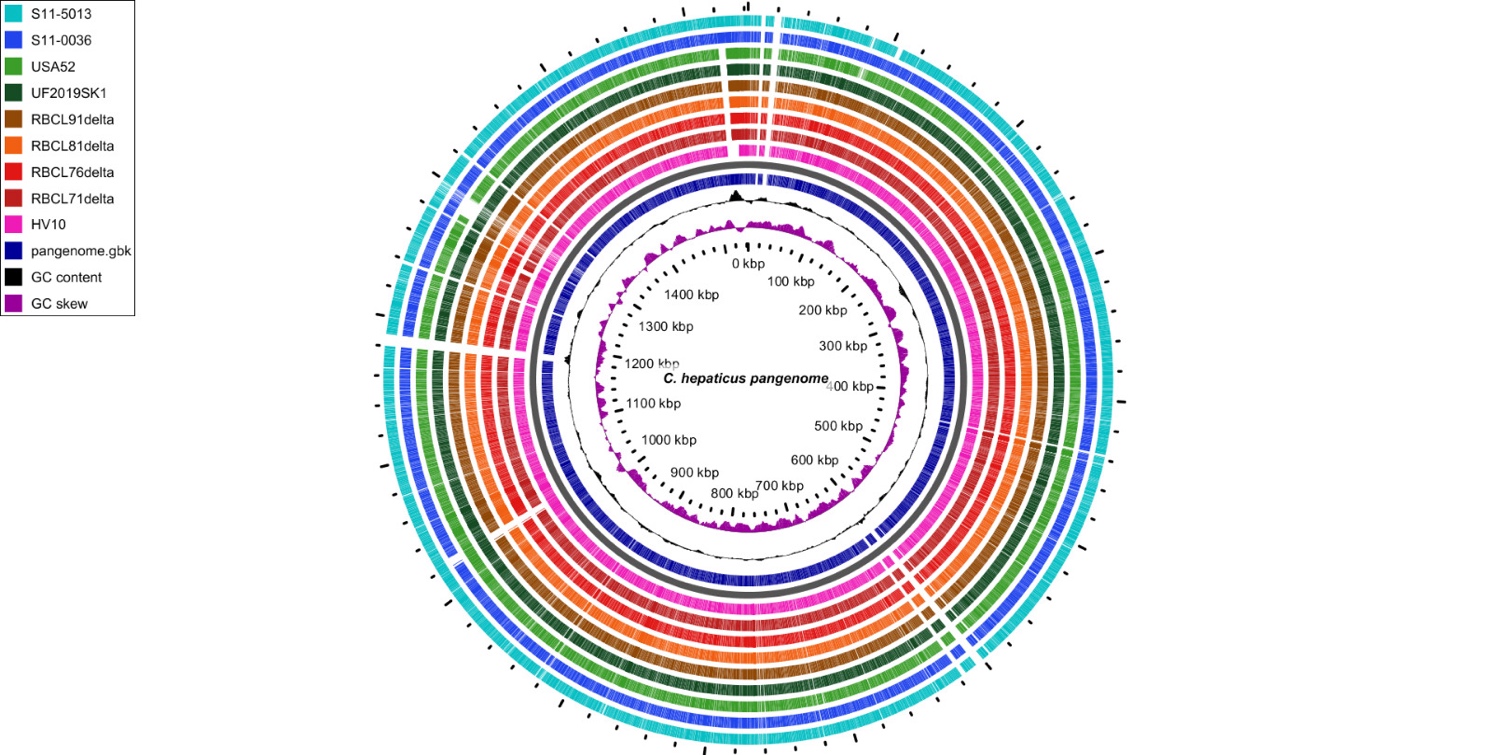


**Supplementary Figure S3.** Pan Genome of 9 *C. hepaticus* genomes from different countries (USA, UK, and Australia).

**Supplementary Figure S4.** Proteins in the region next to 1300 kbp in all *C. hepaticus* genomes, except in *C. hepaticus* USA52.


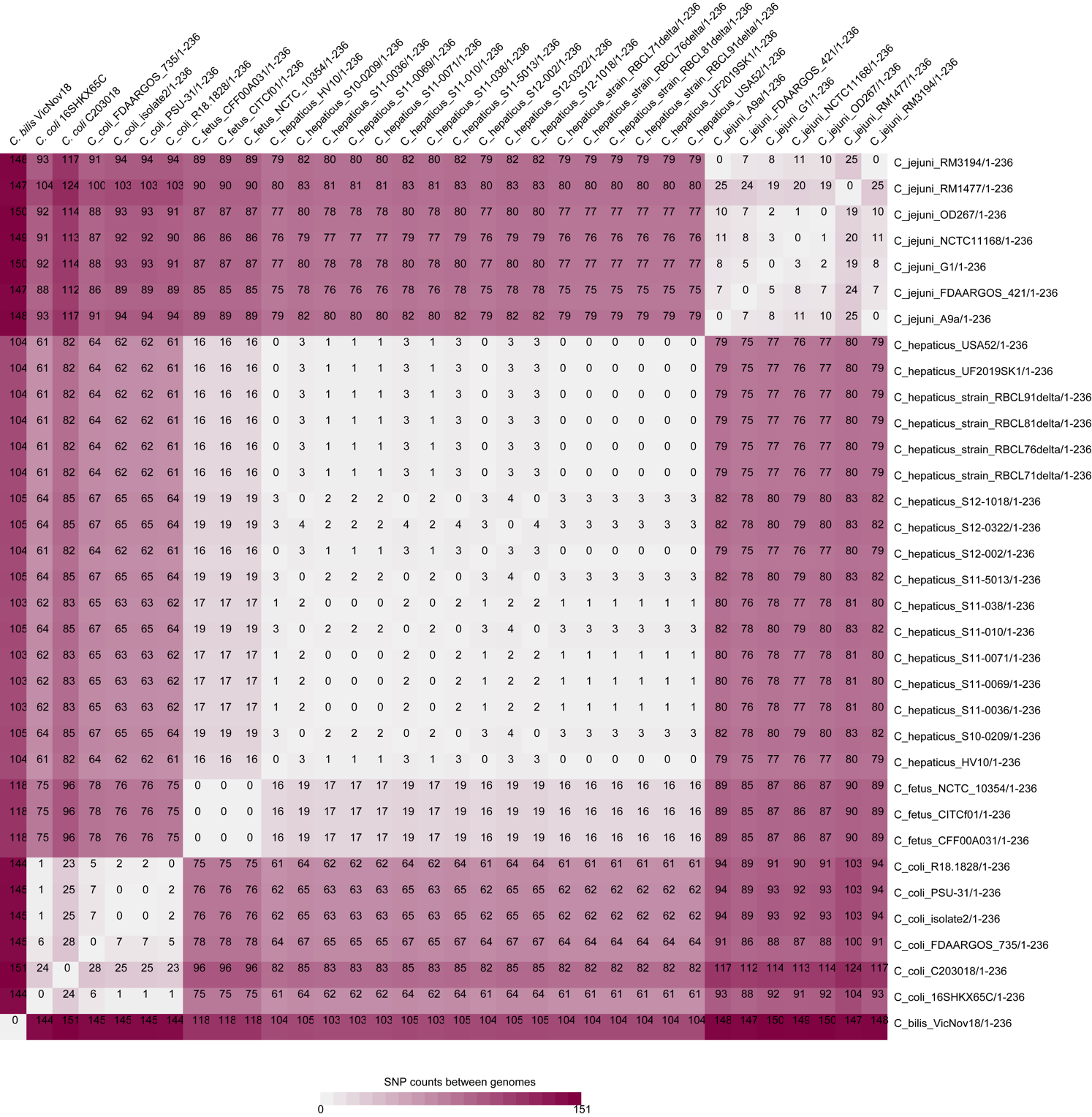


**Supplementary Figure S5:** SNP matrix from whole-genome alignment. Color intensity increases with the difference in SNPs counts between genomes. The minimal difference observed was 0 SNPs
while 151 was the highest.

# Supplementary Tables

**Supplementary Table S1.** Virulence genes identified in the four *C. hepaticus* genomes from Georgia (USA) using ABRicate and VFanalyzer. **1** = detected; **0** = non detected.

|  | **ABRicate** | | | | **VFanalyzer** | | | | |
| --- | --- | --- | --- | --- | --- | --- | --- | --- | --- |
| **Gene** | **RBCL71delta** | **RBCL76delta** | **RBCL81delta** | **RBCL91delta** | **RBCL71delta** | **RBCL76delta** | **RBCL81delta** | **RBCL91delta** |  |
| ***ggt*** | 0 | 0 | 0 | 0 | 1 | 1 | 1 | 1 |  |
| ***cadF*** | 1 | 1 | 1 | 1 | 1 | 1 | 1 | 1 |  |
| ***cheA*** | 1 | 1 | 1 | 1 | 0 | 0 | 0 | 0 |  |
| ***cheV*** | 1 | 1 | 1 | 1 | 0 | 0 | 0 | 0 |  |
| ***cheW*** | 1 | 1 | 1 | 1 | 0 | 0 | 0 | 0 |  |
| ***cheY*** | 1 | 1 | 1 | 1 | 0 | 0 | 0 | 0 |  |
| ***ciaB*** | 1 | 1 | 1 | 1 | 1 | 1 | 1 | 1 |  |
| ***Cj1419c*** | 1 | 1 | 1 | 1 | 0 | 0 | 0 | 0 |  |
| ***Cj1420c*** | 1 | 1 | 1 | 1 | 0 | 0 | 0 | 0 |  |
| ***Cj1427c*** | 1 | 1 | 1 | 1 | 0 | 0 | 0 | 0 |  |
| ***eptC*** | 1 | 1 | 1 | 1 | 0 | 0 | 0 | 0 |  |
| ***flaA*** | 0 | 0 | 0 | 0 | 1 | 1 | 1 | 1 |  |
| ***flaB*** | 0 | 0 | 0 | 0 | 1 | 1 | 1 | 1 |  |
| ***flaG*** | 0 | 0 | 0 | 0 | 1 | 1 | 1 | 1 |  |
| ***flgA*** | 1 | 1 | 1 | 1 | 0 | 0 | 0 | 0 |  |
| ***flgB*** | 1 | 1 | 1 | 1 | 1 | 1 | 1 | 1 |  |
| ***flgC*** | 1 | 1 | 1 | 1 | 1 | 1 | 1 | 1 |  |
| ***flgD*** | 0 | 0 | 0 | 0 | 1 | 1 | 1 | 1 |  |
| ***flgE*** | 1 | 1 | 1 | 1 | 1 | 1 | 1 | 1 |  |
| ***flgE2*** | 0 | 0 | 0 | 0 | 1 | 1 | 1 | 1 |  |
| ***flgF*** | 1 | 1 | 1 | 1 | 0 | 0 | 0 | 0 |  |
| ***flgG*** | 1 | 1 | 1 | 1 | 1 | 1 | 1 | 1 |  |
| ***flgG2*** | 0 | 0 | 0 | 0 | 1 | 1 | 1 | 1 |  |
| ***flgH*** | 1 | 1 | 1 | 1 | 1 | 1 | 1 | 1 |  |
| ***flgI*** | 1 | 1 | 1 | 1 | 1 | 1 | 1 | 1 |  |
| ***flgK*** | 1 | 1 | 1 | 1 | 1 | 1 | 1 | 1 |  |
| ***flgL*** | 0 | 0 | 0 | 0 | 1 | 1 | 1 | 1 |  |
| ***flgM*** | 1 | 1 | 1 | 1 | 0 | 0 | 0 | 0 |  |
| ***flgP*** | 1 | 1 | 1 | 1 | 0 | 0 | 0 | 0 |  |
| ***flgQ*** | 1 | 1 | 1 | 1 | 0 | 0 | 0 | 0 |  |
| ***flgR*** | 1 | 1 | 1 | 1 | 1 | 1 | 1 | 1 |  |
| ***flgS*** | 1 | 1 | 1 | 1 | 0 | 0 | 0 | 0 |  |
| ***flhA*** | 1 | 1 | 1 | 1 | 1 | 1 | 1 | 1 |  |
| ***flhB*** | 1 | 1 | 1 | 1 | 1 | 1 | 1 | 1 |  |
| ***flhF*** | 1 | 1 | 1 | 1 | 1 | 1 | 1 | 1 |  |
| ***flhG*** | 1 | 1 | 1 | 1 | 1 | 1 | 1 | 1 |  |
| ***fliA*** | 1 | 1 | 1 | 1 | 1 | 1 | 1 | 1 |  |
| ***fliD*** | 0 | 0 | 0 | 0 | 1 | 1 | 1 | 1 |  |
| ***fliE*** | 0 | 0 | 0 | 0 | 1 | 1 | 1 | 1 |  |
| ***fliF*** | 1 | 1 | 1 | 1 | 1 | 1 | 1 | 1 |  |
| ***fliG*** | 1 | 1 | 1 | 1 | 1 | 1 | 1 | 1 |  |
| ***fliH*** | 1 | 1 | 1 | 1 | 1 | 1 | 1 | 1 |  |
| ***fliI*** | 1 | 1 | 1 | 1 | 1 | 1 | 1 | 1 |  |
| ***fliL*** | 1 | 1 | 1 | 1 | 1 | 1 | 1 | 1 |  |
| ***fliM*** | 1 | 1 | 1 | 1 | 1 | 1 | 1 | 1 |  |
| ***fliN*** | 1 | 1 | 1 | 1 | 1 | 1 | 1 | 1 |  |
| ***fliP*** | 1 | 1 | 1 | 1 | 1 | 1 | 1 | 1 |  |
| ***fliQ*** | 1 | 1 | 1 | 1 | 1 | 1 | 1 | 1 |  |
| ***fliR*** | 1 | 1 | 1 | 1 | 1 | 1 | 1 | 1 |  |
| ***fliS*** | 1 | 1 | 1 | 1 | 1 | 1 | 1 | 1 |  |
| ***fliW*** | 1 | 1 | 1 | 1 | 0 | 0 | 0 | 0 |  |
| ***fliY*** | 1 | 1 | 1 | 1 | 1 | 1 | 1 | 1 |  |
| ***gmhA*** | 1 | 1 | 1 | 1 | 0 | 0 | 0 | 0 |  |
| ***gmhB*** | 1 | 1 | 1 | 1 | 0 | 0 | 0 | 0 |  |
| ***hddA*** | 1 | 1 | 1 | 1 | 0 | 0 | 0 | 0 |  |
| ***hddC*** | 1 | 1 | 1 | 1 | 0 | 0 | 0 | 0 |  |
| ***hldD*** | 1 | 1 | 1 | 1 | 0 | 0 | 0 | 0 |  |
| ***hldE*** | 1 | 1 | 1 | 1 | 0 | 0 | 0 | 0 |  |
| ***jlpA*** | 0 | 0 | 0 | 0 | 1 | 1 | 1 | 1 |  |
| ***kpsC*** | 1 | 1 | 1 | 1 | 0 | 0 | 0 | 0 |  |
| ***kpsD*** | 1 | 1 | 1 | 1 | 0 | 0 | 0 | 0 |  |
| ***kpsE*** | 1 | 1 | 1 | 1 | 0 | 0 | 0 | 0 |  |
| ***kpsF*** | 1 | 1 | 1 | 1 | 0 | 0 | 0 | 0 |  |
| ***kpsM*** | 1 | 1 | 1 | 1 | 0 | 0 | 0 | 0 |  |
| ***kpsS*** | 1 | 1 | 1 | 1 | 0 | 0 | 0 | 0 |  |
| ***kpsT*** | 1 | 1 | 1 | 1 | 0 | 0 | 0 | 0 |  |
| ***maf3*** | 0 | 0 | 0 | 0 | 1 | 1 | 1 | 1 |  |
| ***maf7*** | 0 | 0 | 0 | 0 | 1 | 1 | 1 | 1 |  |
| ***motA*** | 1 | 1 | 1 | 1 | 1 | 1 | 1 | 1 |  |
| ***motB*** | 1 | 1 | 1 | 1 | 1 | 1 | 1 | 1 |  |
| ***pebA*** | 1 | 1 | 1 | 1 | 1 | 1 | 1 | 1 |  |
| ***pflA*** | 1 | 1 | 1 | 1 | 1 | 1 | 1 | 1 |  |
| ***pgi*** | 0 | 0 | 0 | 0 | 0 | 0 | 1 | 0 |  |
| ***pglA*** | 0 | 0 | 0 | 0 | 1 | 1 | 1 | 1 |  |
| ***pglB*** | 0 | 0 | 0 | 0 | 1 | 1 | 1 | 1 |  |
| ***pglC*** | 0 | 0 | 0 | 0 | 1 | 1 | 1 | 1 |  |
| ***pglD*** | 0 | 0 | 0 | 0 | 1 | 1 | 1 | 1 |  |
| ***pglE*** | 0 | 0 | 0 | 0 | 1 | 1 | 1 | 1 |  |
| ***pglF*** | 0 | 0 | 0 | 0 | 1 | 1 | 1 | 1 |  |
| ***pglG*** | 0 | 0 | 0 | 0 | 1 | 1 | 1 | 1 |  |
| ***pglH*** | 0 | 0 | 0 | 0 | 1 | 1 | 1 | 1 |  |
| ***pglI*** | 0 | 0 | 0 | 0 | 1 | 1 | 1 | 1 |  |
| ***pglJ*** | 0 | 0 | 0 | 0 | 1 | 1 | 1 | 1 |  |
| ***porA*** | 0 | 0 | 0 | 0 | 1 | 1 | 1 | 1 |  |
| ***pseA*** | 1 | 1 | 1 | 1 | 1 | 1 | 1 | 1 |  |
| ***pseB*** | 1 | 1 | 1 | 1 | 1 | 1 | 1 | 1 |  |
| ***pseC*** | 1 | 1 | 1 | 1 | 1 | 1 | 1 | 1 |  |
| ***pseD/maf2*** | 0 | 0 | 0 | 0 | 1 | 1 | 1 | 1 |  |
| ***pseE/maf5*** | 0 | 0 | 0 | 0 | 1 | 1 | 1 | 1 |  |
| ***pseF*** | 1 | 1 | 1 | 1 | 1 | 1 | 1 | 1 |  |
| ***pseG*** | 1 | 1 | 1 | 1 | 1 | 0 | 0 | 1 |  |
| ***pseH*** | 1 | 1 | 1 | 1 | 1 | 1 | 1 | 1 |  |
| ***pseI*** | 1 | 1 | 1 | 1 | 1 | 1 | 1 | 1 |  |
| ***rfbC*** | 1 | 1 | 1 | 1 | 0 | 0 | 0 | 0 |  |
| ***rpoN*** | 1 | 1 | 1 | 1 | 0 | 0 | 0 | 0 |  |
| ***waaC*** | 1 | 1 | 1 | 1 | 0 | 0 | 0 | 0 |  |
| ***waaV*** | 1 | 1 | 1 | 1 | 0 | 0 | 0 | 0 |  |

**Supplementary Table S2.** BLAST statistics of *C. hepaticus* genomes against the reference genome, *C. hepaticus* HV10.

| **Genomes against HV10** | **Per. ID (%)** | **Query cover (%)** | **Max Score** | **Total Score** |
| --- | --- | --- | --- | --- |
| RBCL71delta | 99.95 | 99 | 5.47E+05 | 3.27E+06 |
| RBCL76delta | 99.95 | 99 | 5.47E+05 | 3.54E+06 |
| RBCL81delta | 99.95 | 99 | 5.47E+05 | 3.46E+06 |
| RBCL91delta | 99.95 | 99 | 5.47E+05 | 3.46E+06 |
| S10-0209 | 99.93 | 99 | 2.42E+05 | 2.85E+06 |
| S11-010 | 99.87 | 99 | 2.67E+05 | 2.85E+06 |
| S11-0036 | 99.97 | 99 | 2.71E+05 | 2.84E+06 |
| S11-0038 | 99.91 | 99 | 3.00E+05 | 2.84E+06 |
| S11-0069 | 99.92 | 99 | 3.00E+05 | 2.87E+06 |
| S11-0071 | 99.91 | 99 | 3.00E+05 | 2.86E+06 |
| S11-5013 | 99.87 | 99 | 2.67E+05 | 2.83E+06 |
| S12-002 | 99.91 | 99 | 3.33E+05 | 2.81E+06 |
| S12-0322 | 99.92 | 98 | 2.41E+05 | 2.82E+06 |
| S12-1018 | 99.87 | 99 | 2.67E+05 | 2.84E+06 |
| UF2019SK1 | 99.92 | 99 | 8.88E+05 | 3.33E+06 |
| USA52 | 99.8 | 98 | 4.51E+05 | 3.24E+06 |
| **Average** | **99.91** | **98.87** | **3.95E+05** | **3.04E+06** |
